# Lymphatic filariasis elimination status: *Wuchereria bancrofti* infections in human populations and factors contributing to continued transmission after seven rounds of mass drug administration in Masasi District, Tanzania

**DOI:** 10.1371/journal.pone.0262693

**Published:** 2022-01-19

**Authors:** Eliza T. Lupenza, Dinah B. Gasarasi, Omary M. Minzi

**Affiliations:** 1 Department of Parasitology and Medical Entomology, School of Public health and Social Sciences, Muhimbili University of Health and Allied Sciences, Dar es Salaam, Tanzania; 2 Department of Clinical Pharmacy, School of Pharmacy, Muhimbili University of Health and Allied Sciences, Dar es Salaam, Tanzania; Instituto Rene Rachou, BRAZIL

## Abstract

**Background:**

Lymphatic filariasis (LF) affects more than 120 million people globally. In Tanzania, nearly six million people are estimated to live with clinical manifestations of the disease. The National LF control program was established in 2000 using Mass drug administration (MDA) of Ivermectin and Albendazole to individuals aged 5years and above. This study assessed the infection status in individuals aged 15 years and above who are eligible for participation in MDA. The level of compliance to MDA and the reasons for non-compliance to MDA were also assessed.

**Methods:**

A community based cross-sectional study was conducted in two villages of Masasi District. A total of 590 participants aged 15 years and above were screened for the circulating filarial antigen (CFA) using the rapid diagnostic test. Night blood samples from CFA positive individuals were further analyzed for detection and quantification of *Wuchereria bancrofti* microfilaria (Mf) using the counting chamber technique. A pre-tested questionnaire was administered to collect information on compliance to MDA and the factors affecting continued transmission. Data were analyzed using SPSS Version 20. Chi-square test was used to compare the prevalence of CFA by gender and village where a *P*-value ≤0.05 was considered statistically significant.

**Results:**

Out of 590 participants, 30 (5.1%) were positive for CFA and one (0.2%) was found positive for microfilaria of *Wuchereria bancrofti*. Compliance during the last round of MDA, in the year 2019 was 56% which is below the minimum coverage recommended by WHO. Absence from home during MDA and perceptions of being free from hydrocele or elephantiasis were the major reasons for non-compliance.

**Conclusion:**

There is a significant decline in LF transmission in Masasi District after seven rounds of MDA. However, the presence of individuals who are persistently non-compliant may delay elimination of LF in the District.

## Background

Lymphatic filariasis (LF) due to infections with *Wuchereria bancrofti* is one of the major vector-borne diseases in sub-Saharan Africa and the second leading cause of disability globally due to lymphoedema, elephantiasis and hydrocele [1]. LF affects more than 120 million people globally with about 40 million disfigured and incapacitated by the disease [2]. In Tanzania nearly six million people are estimated to live with debilitating manifestations of the disease [3]. Adult worms nest in the lymphatic vessels and disrupt the normal function of the lymphatic system. The worms can live for approximately 6–8 years and, during their lifetime, produce millions of microfilaria (immature larvae) that circulate in the blood and are infective to the vector mosquitoes [4].

Recognizing the economic impact, disability and social stigma associated with LF and the availability of strategies to prevent infection and manage morbidity; the World Health Organization (WHO) initiated the global program for elimination of Lymphatic filariasis (GPELF) in the year 2000 [5]. The strategy was based on mass drug administration of preventive chemotherapy using combination therapy of Ivermectin (IVM) or Diethylcarbamazin citrate (DEC) with Albendazole (ALB) and morbidity management for those already affected [5].

The combination of IVM plus ALB is used in areas of Africa where Onchocerciasis (river blindness) is co-endemic with LF [6] because, DEC is known to have side effects on Onchocerciasis patients such as dizziness, nausea, fever, headache, immediate hypersensivity reactions and muscles or joint pains [7].

MDA treatment was advocated because it reduces the density of microfilaria and the prevalence of infection in the community. Maintaining microfilaria density to very low levels with annual MDA was believed to eventually lead to the elimination of the infection [8]. Furthermore, after six rounds of MDA with coverage of 65% to 70% of the target population it was estimated that transmission would be completely interrupted [9].

Indeed, there have been reports on global decline of LF transmission since the initiation of MDA programs [10,11]; subsequently, sixteen countries are now acknowledged to have achieved elimination of lymphatic filariasis as a public health problem [4]. In addition, WHO reported that, about 597 million people no longer require preventive chemotherapy [4].

Despite these successes there have been reports of persistent transmission in some areas with the on-going MDA over a decade [12–14]. In Ghana for instance, 14 rounds of MDA did not stop the transmission of LF in districts with relatively high baseline prevalence [12]; while in Mafia Islands, Tanzania the evidence of LF continued transmission was reported after 15 rounds of MDA [14]. The continued transmission of LF is linked to low drug uptake [15–17], the presence of epidemiological hotspots and systematic non-compliant individuals who potentially serve as reservoirs of infection [18]. The compliance to MDA is however, known to be affected by a number of factors including; fear of side effects, a general dislike of taking drugs, low motivation of drug distributors, lack of knowledge of the disease in question and inadequate communication on the rationale of MDA as previously reported [15,19–22].

In Masasi district, LF elimination activities began in the year 2012, with consecutive MDA from 2012 to 2019. During this period, the reported drug treatment coverage was generally higher, ranging between 92% and 93% (National NTDs report, unpublished). A recent study reports low (0.5%) infection rate in vector populations [23], however, there is no published information for Masasi District regarding the current status of *W*. *bancrofti* infection in the human population. Therefore, this study was designed to assess the infection status in the human population, compliance to MDA and reasons for non-compliance to MDA.

## Materials and methods

### Study area

The study was conducted in Masasi district council (10.7348° S, 38.8044° E) Southeastern Tanzania. The total population of the district is 247,993 [24], while the population of Mbuyuni and Maparagwe villages was 5,667 and 1,185 respectively. The district is endemic for LF and had a prevalence of CFA of 11.7% prior to the MDA campaigns, launched in the year 2012 as previously described [23]. LF elimination in the District is based on MDA using Ivermectin and Albendazole for individuals aged 5years and above.

### Study design and selection of study participants

A community based cross-sectional study was conducted in August 2020 in Maparagwe and Mbuyuni villages. The study covered a total of 11 hamlets: Maparagwe has five hamlets while Mbuyuni has six hamlets. A sample size of 502 was considered sufficient to estimate the community prevalence of CFA of 5% using the previously established sample size formula [25]. A 95% level of confidence and a 2% absolute precision was used and adjustment for an anticipated non-response rate of 10% was made. Convenience sampling methods were used to recruit participants aged 15 years and above, this sub-population included those individuals who have participated in the MDA for the past seven rounds.

Prior to commencing the study; meetings were conducted with the village leadership in each respective village; during these meetings the purpose of the study and methods were clarified. When the research activities were initiated, community members were mobilized through village leaders and gathered in a defined area.

### Estimating the prevalence of *W*. *bancrofti* circulating filarial antigen

A total of 590 participants were screened for circulating filarial antigen (CFA). Seventy-five micro liters (75μl) of blood sample was taken by finger-pricking, then slowly added onto Alere^™^ Filariasis Test Strip (FTS) (Alere©, Waltham, United States), following the manufacturer’s instructions. Briefly, the test cards were placed on a table and left for 15 minutes before reading the results. For CFA positive samples, a second 75μl blood sample obtained by finger-prick was collected for a confirmatory test as described above.

### Identification and quantification of *W*. *bancrofti* microfilaria

For individuals with CFA positive test results, night blood samples were collected between 22:00 and 00:00hrs using finger-prick method. Briefly 100μl of blood was taken from each participant using sterile heparinized capillary tubes and transferred into a vial containing 1ml of 3% acetic acid. Each haemolysed sample was microscopically examined for microfilaria using the counting chamber technique at 10× magnification. The microfilaria density was quantitated and expressed as number of Mf per 100 microliters of blood. Double microscopic reading was done for quality assurance of the data.

### Questionnaire survey

A pre-tested, structured questionnaire was administered to 588 participants, social demographic data including; age, sex, education, occupation and the duration of stay in Masasi district were recorded. Participants were also interviewed on whether they ever participated in previous MDAs. Those who never participated in any MDA or did not swallow the tablets were asked to provide the reasons. The official programme coverage for Masasi District Council was obtained from the District NLFEP office.

### Data analysis

Data were entered into the computer and analyzed using statistical package for social sciences (SPSS) Version 20.0 (SPSS, Inc., IL, USA). Independent variables included; age, sex and study village. The dependent variables included CFA and Mf status. Descriptive statistics was done to summarize demographic information of the participants, estimate the prevalence of CFA, Mf and the reasons for non-compliance to MDA. The comparison of CFA prevalence by sex and village was done using chi-square test and the significance level was set at *P* ≤0.05 with a 95% confidence level. The density of microfilaria was estimated as number of microfilaria per 100 microliters of blood.

### Ethical statement

Ethical clearance for the study was provided by the Research and publications Committee of Muhimbili University of Health and Allied sciences, Tanzania (IRB.NO.MUHAS-REC-9-2019-053). The permission to conduct the study was obtained from the District medical officer and the local government leadership of the respective village. Prior to the study, meetings were held with local government leadership in each respective village and the aim of the study was clarified. All participants were asked to give written informed consent prior to interviews and CFA testing. For the participants below age of 18 years, consents were obtained from parents/guardians. Additional assents were sought from the specific participants to ensure their voluntary participation.

## Results

### Social demographic characteristics of the study participants

A total of 590 individuals from Maparagwe and Mbuyuni villages participated in the study. Of these 63.6% (375/590) were female. The age distribution of the respondents ranged from 15 to 96 years (mean = 49.4years; median = 50years). A total of 90.2% (532/590) of the respondents were farmers and 72.2% had primary education. More than 90% of the respondents had stayed in the village for more than 5years (Table 1).

**Table 1:** Social demographic characteristics of the study participants.

Characteristics	No. of people (%) N = 590
**Gender**	
Male	215 (36.4)
Female	375 (63.6)
**Age group**	
15–25	62 (10.5)
26–36	87 (14.7)
37–47	120 (20.3)
48–58	132 (22.4)
59–69	96 (16.3)
70 and above	93 (15.8)
**Marital status**	
Single/not married	185 (31.4)
married	405(68.6)
**Village**	
Mbuyuni	355(60.2)
Maparagwe	235 (39.8)
**Education**	
Primary education	426 (72.2)
Secondary education	28 (4.7)
Post-secondary/College/University	7 (1.2)
Not gone to school	129 (21.9)
**Occupation**	
Student	3(0.5)
Farmer/peasant	532(90.1)
Business	24 (4.1)
Public/private employee	5(0.8)
No occupation	16 (2.7)
Other occupations	10 (1.7)
**Length of stay in the village**	
1 to 6months	5 (0.8)
6months to 1year	1(0.2)
1 to 5years	27 (4.6)
More than 5 years	557 (94.4)

### Prevalence of CFA and Microfilaria in the study community

Of the 590 participants screened for CFA, 5.1% (30/590) were found positive (95% CI, 4.1–5.7). The differences of CFA prevalence by gender, age group and villages, was not statistically significant (*P*>0.05). Analysis of night blood samples from 20 CFA positive individuals using the counting chamber technique found only one (5%) Mf positive individual and the Mf prevalence was 0.2% (Table 2).

**Table 2:** Prevalence of CFA and microfilariae infection by gender, age group and village.

Characteristics	No.(%) of people examined	No.(%)with CFA	95% CI (Lower-upper)	*P*-value	No.(%) with MF
**Gender**					
Male	215 (36.4)	12 (5.6)	5.3–6.0	0.68	1 (0.5)
Female	375 (63.6)	18 (4.8)	4.4–5.2		0 (0.0)
Total	590	30 (5.1)	4.1–5.7		1 (0.2)
**Age group**					
15–25	62 (10.5)	1 (1.6)	1.36–1.84		0
26–36	87 (14.7)	2 (2.3)	2.1–2.5		0
37–47	120 (20.3)	4 (3.3)	2.9–3.7		0
48–58	132 (22.4)	13 (9.8)	8.3–11.3	0.15	0
59–69	96 (16.3)	4 (4.2)	3.6–4.8		1 (1.04)
70 and above	93 (15.8)	6(6.5)	5.3–7.7		0
Total	590	30 (5.1)	4.1–5.7		1(0.2)
**Village**					
Mbuyuni	355 (60.2)	19 (5.4)	5.1–5.7	0.72	0 (0.0)
Maparagwe	235 (39.8)	11 (4.7)	4.4–5.0		1(0.43)
**Total**	590	30 (5.1)	4.1–5.7		1(0.2)

### MDA coverage and compliance in the study community

The reported MDA coverage reveals high treatment coverage for the past three years ranging from 92% to 93% which is above 65%, the recommended minimum coverage by WHO [8] (Table 3).

**Table 3:** Reported treatment coverage for MDA with Ivermectin and Albendazole in the past three years in Masasi District.

Treatment year	Targeted Population	No. of people Treated (% coverage)
2016	237,113	221,474 (93.3)
2017	277,268	259,367 (93.4)
2018	57,117	52,749 (92.3)

Source: Tanzania Neglected tropical disease control programme.

A questionnaire survey of 588 participants revealed that 78.1% (n = 459) had participated in MDA campaigns by receiving the tablets in at least one round of MDA and only 3% (13/459) didn’t swallow the tablets. More than a half, 58% (339/588) participated in the last round of MDA conducted in September 2019, about 10 months before this study. The overall compliance was 56% (n = 330) (Table 4). More than 20% (129/588) of the respondents admitted having never participated or swallowed the tablets in any rounds of MDA; these individuals are referred to as systematic non-compliants. The proportion of systematic non-compliant was significantly higher in Mbuyuni compared to Maparagwe village (*χ2* = 4.612, df = 1, *P* = 0.032) (Table 4). Furthermore, the level of compliance during previous year’s (2019) MDA was significantly low in Mbuyuni compared to Maparagwe village (*χ2* = 8.429, df = 1, *P* = 0.004). The level of compliance among females and males was 60.3% and 48.8% respectively (Table 4). The CFA positivity among systematic non-compliant was 4.7% (6/129) vs. 5.4% (24/447) among participants who had participated during previous MDA rounds (*X*^*2*^ = *0*.275, *P* = 0.67).

**Table 4:** Level of compliance to taking Ivermectin and Albendazole during MDA by gender and village of residence.

Characteristics	Compliance during 2019 MDA round (%)	*X* ^*2*^ *-value (P-value)*	Complied in at least one previous MDA round (%)	*X* ^*2*^ *-value (P-value)*	Systematic non-compliance	*X* ^*2*^ *-value (P-value)*
**Gender**						
Females (n = 375)	226 (60.3)	7.220 (0.007)	294(78.4)	1.611 (0.204)	71 (18.9)	
Males (n = 213)	104 (48.8)		153 (71.8)		58 (27.2)	5.460 (0.019)
**Village**						
Maparagwe (n = 235)	149(63.4)	8.429 (0.004)	190 (80.9)	0.003 (0.954)	41 (17.4)	
Mbuyuni (n = 353)	181 (52.4)		257 (72.8)		88 (24.9)	4.612 (0.032)
**Overall compliance (n = 588**	**330 (56.1)**		**447 (76)**		**129 (21.9)**	

### Reasons for non-compliance to MDA campaigns

**Fig 1:**
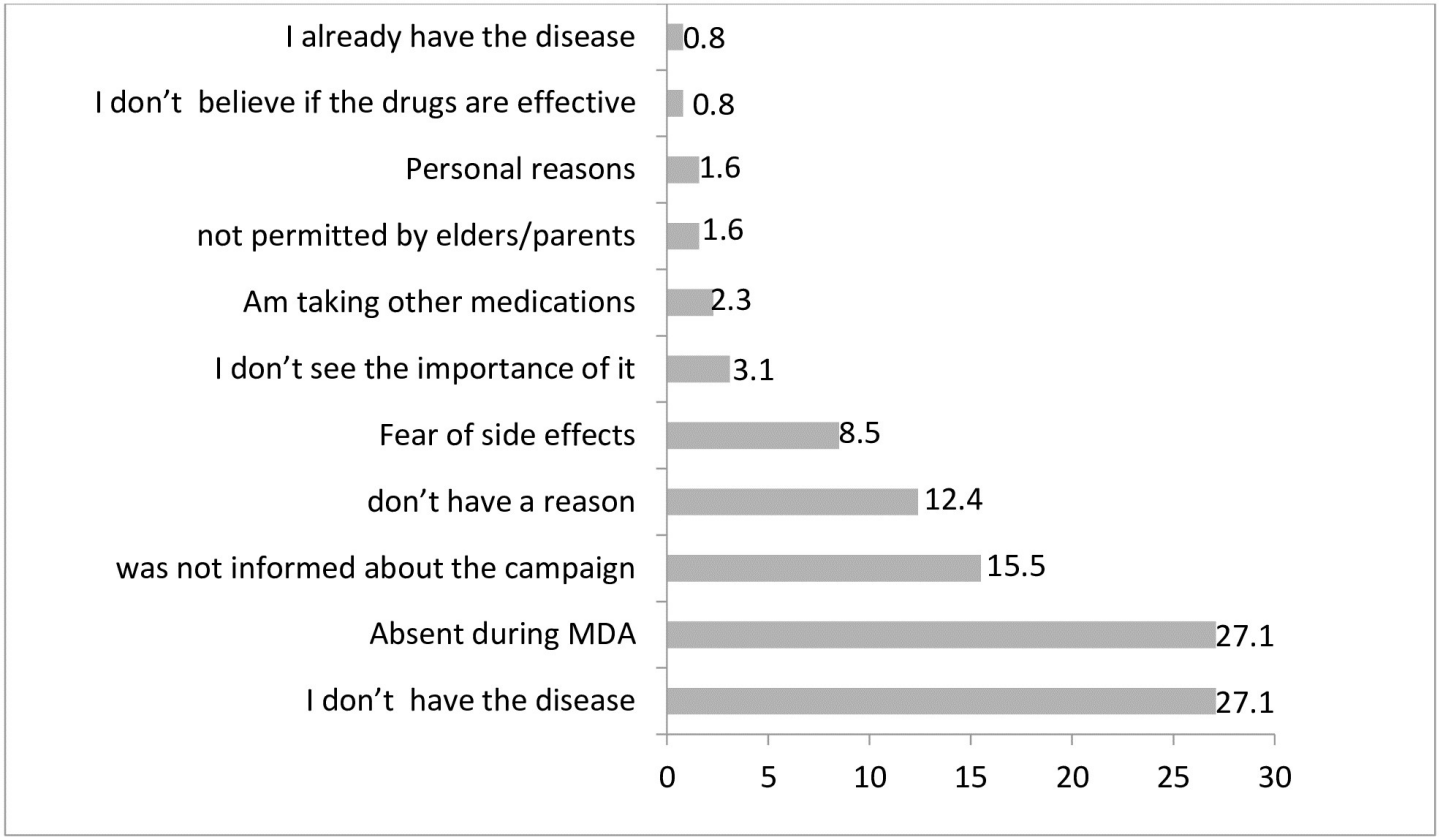
Percent (%) response on reasons for non-compliance to MDA (n = 129).

Of the 129 participants who never participated in any round of MDA, 27.1% (n = 35) admitted being absent during the time of MDA and a similar proportion, perceived that they did not have the disease hence no need to participate. More than 15% (n = 20) reported that they were not informed about the campaign, 8.5% (n = 11) feared the side effects related to the drugs while more than 12% had no specific reasons (Fig 1).

## Discussion

The current study reports the CFA and Mf prevalence of 5.1% and 0.2% respectively, in the human population after seven rounds of MDA. At baseline (year 2012) the prevalence of CFA was 11.7% [National NTD report, 2012]. Compared to the current findings, this indicates a nearly 50% reduction of parasite antigenemia.

The prevalence of Mf was below the WHO-recommended elimination threshold of 1%; however, the prevalence of antigenemia remains above the recommended threshold of 2% [6], indicating that the district is yet to interrupt LF transmission. The prevalence of CFA and Mf reported in the current study are corroborated by the findings from a recent study in Mkinga District, North-eastern Tanzania [26] reporting a CFA and Mf prevalence of 5.8% and 0.3% respectively. Similarly, a significant drop in microfilaraemia was reported in Zanzibar after five rounds of MDA with ivermectin and albendazole complemented with intensive community mobilization and high treatment coverage [27].

Treatment coverage and compliance are crucial factors to achieve LF elimination [28]. The reported coverage in the district was significantly higher, ranging from 92% to 93%; however, the compliance during previous year’s (2019) MDA, 10 months before the present study, was 56% which is below the WHO recommended minimum coverage of 65% [8]. This may indicate a coverage–compliance gap in the district (the proportion of people who receive the drugs but do not ingest them) as reported in some studies [29,30]. Although, a minority, of the respondents admitted having received the tablets but did not swallow them. The reasons for not swallowing the tablets were related to the fear of side effects, taking other medications and not seeing the importance because they do not have the disease.

In addition, the low compliance recorded in this study may be due to, lack of clear understanding about MDA. For instance, nearly 22% of individuals who did not comply with MDA perceived that, people with hydrocele or elephantiasis are the ones who should swallow the drugs. These findings are similar to those of a recent study in Northern Tanzania, where about 40% of the study participants did not take drugs during the previous MDA [27]. Thus, low compliance could be one of the reasons for persistent transmission of LF in the district. For instance, a study in Rufiji District reports higher prevalence of CFA and hydrocele among men who did not take the drugs in the previous MDA [15].

In addition a spatial heterogeneity in compliance to MDA was observed between the two study villages; Mbuyuni had a significantly low compliance level during the previous year’s (2019) MDA and a higher proportion of systematic non-compliants compared to Maparagwe village. It has also been established that, individuals who are persistently non-compliant to MDA campaigns serve as reservoirs of infection to mosquito vectors [18]. The heterogeneity in compliance may explain the observed differences in CFA prevalence between the two study villages and it may be hypothesized that non-compliance is a major factor for the continued transmission of LF in this area. Furthermore, compliance was significantly low in male participants compared to females. These findings are corroborated by a previous study in the same region [15]. Non-compliance among male participants could be explained by the fact that majority of men engage in different socio-economic activities outside the home, hence they are mostly away from home during MDA.

Absenteeism at the time of drug distribution was the main reason for non-compliance in this study; surprisingly, it is commonly reported even by persons who are not migrants [15,16,22,31,32]. Based on this fact it seems that the time allocated for MDA and for mopping-up activities is sometimes not sufficient to reach those people who were not found at home at the time of drug distribution. But they could be reached if given sufficient time [21]. The fear of side effects was also reported by a minority (8.5%) of respondents; these findings are corroborated by findings from previous studies [15,16]. Some participants admitted that, they do not have any disease (LF) so they do not see the need to swallow the drugs; it seems there are still misconceptions regarding MDA among community members. Pre-MDA information and communication needs to be emphasized as it may help to dispel some of the misconceptions.

MDAs aim is to reduce Mf density in human populations to a low level that transmission by vectors cannot occur; and eventually interrupt transmission [8]. A low Mf prevalence was reported in this study and it is likely that the observed decline in LF transmission may be attributable to the effect of treatment However, the low Mf prevalence reported could be an underestimate because, more than 30% CFA positive individuals did not consent for night blood collection despite being informed. Furthermore, the use of convenience sampling method to recruit the study participants may have left out some individuals, especially the systematic non-complaints to MDA. In addition, the fact that the study was conducted 10 months after the last round of MDA may have introduced a recall bias in assessing compliance to MDA. Finally, only two villages were included in the study, hence the CFA prevalence reported may not be generalized to the whole district.

## Conclusion

The findings of this study show a decline in prevalence of parasite antigenemia from 11.7% at baseline (year, 2012) to 5.1% after seven rounds of MDA. However, low compliance to MDA, and the presence of individuals who are persistently non-compliant may delay LF elimination in the district. The National NTDs control program should identify individuals who are systematic non-compliant and set up a specific orientation program for them, to bring them at par with the rest of the community; then monitor their participation in the MDA treatment program. In addition, effective information communication on MDA program should be implemented in order to improve compliance and subsequently achieve the elimination target.

## Abbreviations

ALB: Albendazole
CFA: circulating filarial antigens
DEC: diethylcarbamazine citrate
FTS: Filarial test strips
GPELF: Global Programme to Eliminate Lymphatic Filariasis
IVM: ivermectin
LF: Lymphatic filariasis
MDA: mass drug administration
MF: microfilaria
NTDs: Neglected Tropical Diseases
SPSS: Statistical package for social sciences
WHO: World Health Organization
